# Volumetric Bone Mineral Density Measured by HR-pQCT in Patients with Psoriasis or Psoriatic Arthritis: A Systematic Review and Meta-Analysis with Trial Sequential Analysis

**DOI:** 10.3390/healthcare9081056

**Published:** 2021-08-17

**Authors:** Yu-Wen Huang, Jing-Wun Lu, Tai-Li Chen

**Affiliations:** 1 Department of Medical Education, Taipei Tzu Chi Hospital, Buddhist Tzu Chi Medical Foundation, New Taipei City 231, Taiwan; dr.yuwenhuang@gmail.com; 2 Department of Physical Medicine and Rehabilitation, Hualien Tzu Chi Hospital, Buddhist Tzu Chi Medical Foundation, Hualien 970, Taiwan; jingwunlu@gmail.com; 3 Department of Medical Education, Medical Administration Office, Hualien Tzu Chi Hospital, Buddhist Tzu Chi Medical Foundation, Hualien 970, Taiwan; 4 School of Medicine, Tzu Chi University, Hualien 970, Taiwan; 5 Tzu Chi Skin Institute, Hualien Tzu Chi Hospital, Buddhist Tzu Chi Medical Foundation, Hualien 970, Taiwan

**Keywords:** bone density, osteoporosis, volumetric bone mineral density, arthritis, psoriasis, psoriatic arthritis, HR-pQCT, meta-analysis, trial sequential analysis

## Abstract

Bone health in psoriasis and psoriatic arthritis has been emphasized in recent years. Novel imaging modalities allow investigations into volumetric bone mineral density (vBMD) and bone microstructure in psoriatic patients. However, literature regarding vBMD measured by high-resolution peripheral quantitative computed tomography (HR-pQCT) is inconclusive. We conducted a systematic review and meta-analysis to evaluate vBMD in patients with psoriatic disease. We searched PubMed, EMBASE, Web of Science, and Cochrane Library for relevant observational studies. A random-effects meta-analysis with trial sequential analysis (TSA) was performed. The pooled mean difference (MD) and 95% confidence interval (CI) were calculated. Five studies with 780 patients were included. Patients with psoriatic disease showed a lower average vBMD than controls (MD −14.90; 95% CI −22.90 to −6.89; TSA-adjusted CI −23.77 to −6.03; I^2^ = 41%). Trabecular vBMD and cortical vBMD results were inconclusive because of the small sample size. Patients recruited in Asia and those whose vBMD were measured at the distal radius exhibited a lower average vBMD than controls. Further research should clarify the association of psoriasis with bone microstructure and the underlying pathophysiology.

## 1. Introduction

Psoriasis is a chronic inflammatory disorder characterized by papulosquamous lesions with variable morphology, severity, and clinical course [1,2]. While skin involvement is often the most prominent manifestation, recognizing the condition as a chronic, multisystemic disease is imperative to optimize management [3]. Cutaneous psoriasis and psoriatic arthritis (PsA) are recognized in the spectrum of psoriatic disease based on the common pathophysiological process identified in the affected skin and joints [4,5,6]. Hence, considering combination of patients with cutaneous psoriasis and PsA in disease management is clinically reasonable [7,8].

In recent experimental and clinical research, abnormal bone remodeling and new bone formation in psoriatic disease have turned research attention to bone health in psoriatic comorbidities [9,10]. A previous study concluded that psoriatic patients have a higher fracture risk than non-psoriatic controls, without necessary association with lower bone mineral density (BMD) [11]. BMD defined by the commonly used dual-energy X-ray absorptiometry (DXA), which only measures areal BMD (aBMD), may not reflect the actual bone strength [12]. Bone microarchitecture, geometry, and mineralization may also determine the bone strength [13]. The authors hypothesized that low volumetric bone mineral density (vBMD), which is related to low bone quality, is associated with a higher risk of fracture in psoriatic patients.

With the recent developments in imaging modalities, high-resolution peripheral quantitative computed tomography (HR-pQCT) has been introduced to measure bone microarchitecture, bone erosions, and vBMD [14,15]. Persons with normal aBMD measured by DXA may have abnormal vBMD under HR-pQCT [16,17]. HR-pQCT provides three-dimensional images with high resolution, with only low levels of radiation exposure [18], thus enabling studies to investigate vBMD and bone microstructure in psoriatic patients. Nevertheless, the literature regarding vBMD measured by HR-pQCT in psoriatic disease is inconsistent, and the sample sizes of individual studies have been small. To enhance the statistical power and provide a higher level of evidence on this topic, a well-designed systematic review is needed to provide better understanding. Therefore, this systematic review and meta-analysis aimed to assess vBMD measured by HR-pQCT in patients with psoriatic disease.

## 2. Materials and Methods

This meta-analysis was performed based on the Preferred Reporting Items for Systematic reviews and Meta-Analysis [19] and the Meta-Analysis of Observational Studies in Epidemiology guidelines [20]. The methodology was pre-specified in advance and registered on PROSPERO (registration no. CRD42020170873).

### 2.1. Literature Search

Two independent investigators (YWH and JWL) retrieved articles from PubMed, EMBASE, Web of Science, and Cochrane Library databases from database inception until 5 March 2021. A keyword combination of “psoriasis,” “psoriatic arthritis,” “volumetric bone mineral density,” and “bone microstructure,” together with their synonyms and derivatives, was adopted. The details of the search strategy are provided in Table S1. There was no language restriction. Furthermore, reference lists of relevant reviews were manually examined for additional candidates. Discrepancies and disagreements were resolved by discussion with a third author (TLC).

### 2.2. Study Selection and Eligibility

Two investigators (YWH and JWL) independently selected relevant studies according to the following criteria: (1) observational studies (cohort, case-control, or cross-sectional studies) including adults diagnosed with psoriasis or PsA according to clinical or histopathologic information, (2) study involving adult controls without psoriasis or PsA, and (3) studies comprising clinical outcomes of interest, i.e., average vBMD, trabecular vBMD, or cortical vBMD, measured by HR-pQCT. Case reports, letters to editors, and conference abstracts were excluded. No limitations regarding age, sex, or disease severity were applied. Animal studies and studies conducted in laboratory settings were also excluded. Inter-rater reliability was assessed using the Cohen’s *κ* statistic. In case of any disagreements, consensus was achieved by discussion with a third investigator (TLC).

### 2.3. Data Extraction and Outcomes of Interest

Data were collated by two independent investigators (YWH and JWL) from the included studies. The information included the following: first author, year of publication, study design, country, sample size, patient age, sex, body mass index, disease duration, potential osteoporotic/anti-osteoporotic drug usage, and outcomes of interest. The study designs of our included studies were reclassified using the Design Algorithm for Medical Literature [21]. If the effect estimates were insufficient for data analysis, we contact the corresponding authors for relevant information. The primary outcome was the average vBMD. Secondary outcomes were trabecular vBMD and cortical vBMD. Moreover, the Cohen’s *κ* statistic was applied to quantify inter-rater reliability.

### 2.4. Quality Assessment

The risk of bias was evaluated using the Newcastle–Ottawa Scale (NOS) for non-randomized studies [22]. The scale consists of three domains: selection of study groups, comparability of study groups, and ascertainment of the outcome of interest. Two investigators (YWH and JWL) independently evaluated the risk of bias of the eligible studies. Disagreements were resolved by consultation with a third investigator (TLC).

### 2.5. Statistical Analysis

The meta-analysis was conducted with Review Manager 5.4.1 (Cochrane Collaboration, Oxford, UK). Pooled effect sizes and their corresponding confidence intervals (CIs) were calculated using the random-effects model [23]. The mean difference (MD) was calculated with the 95% CI for continuous outcomes. The I2 statistics were applied to quantify the between-study heterogeneity [23,24]. Heterogeneity was considered low, moderate, and high if I^2^ was <50%, 50–75%, and >75%, respectively. A *p* < 0.05 defined statistical significance. A predefined subgroup analysis regarding the geographic locations and measuring sites was also conducted to determine whether certain study-level factors would influence the pooled estimates.

To minimize type I errors potentially caused by the increased risk of random error when sparse samples are analyzed and significance testing was repeated, as such trial sequential analysis (TSA) was applied using TSA Viewer version 0.9.5.10 beta [25,26]. The required information size (IS) was estimated based on an overall type I error of 5% and a power of 90%.

## 3. Results

### 3.1. Search Results

**Figure 1:**
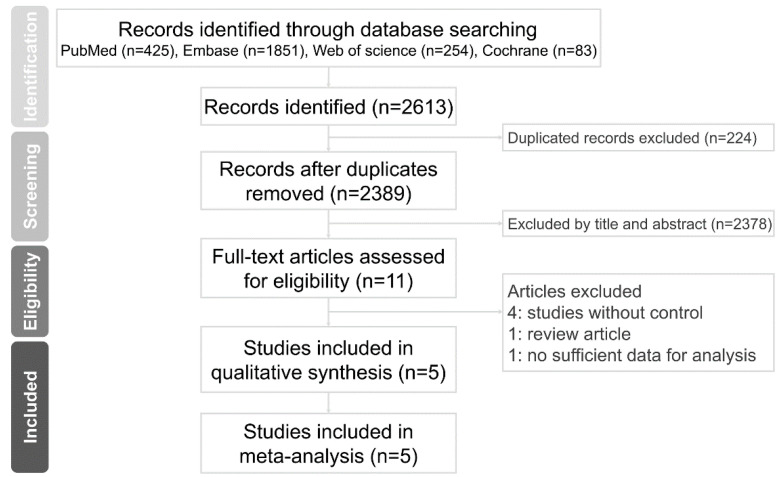
PRISMA flow diagram.

A total of 2613 publications were found on the electronic databases using our search strategy. We identified 2389 articles after removing duplicates, 2378 of which were excluded based on scrutinization of the titles and abstracts. The full text of 11 citations was assessed for eligibility. Of these, six studies were excluded because lack of a control group or the study designs did not fulfill the eligibility criteria. Eventually, five studies were eligible for the final meta-analysis. The PRISMA selection process is illustrated in Figure 1.

### 3.2. Description of Study Characteristics

The baseline characteristics of the five eligible observational studies was summarized in Table 1 [27,28,29,30,31]. The *κ* statistics for study selection and data retrieval were 0.96 and 1, respectively. A total of 780 participants, studied between 2015 and 2020, were evaluated. Among the five included studies, four studies were conducted using a cross-sectional study design [27,28,30,31]. Three studies were conducted in Europe [27,28,29], and the other two were conducted in Asia [30,31]. Potential osteoporotic/anti-osteoporotic drug usage, such as systemic corticosteroids, methotrexate, or anti-tumor necrosis factor-α biologic agents, was identified [32,33,34]. Moreover, vBMD was measured either at the distal radius or at the metacarpal head.

### 3.3. Risk of Bias of the Included Studies

The results of the NOS quality appraisal in each domain are outlined in Figure S1. Four of the included studies were considered of moderate quality. The study by Simon et al. was judged to have “high” quality.

### 3.4. Quantitative Meta-Analysis of the Outcomes of Interest

**Figure 2:**
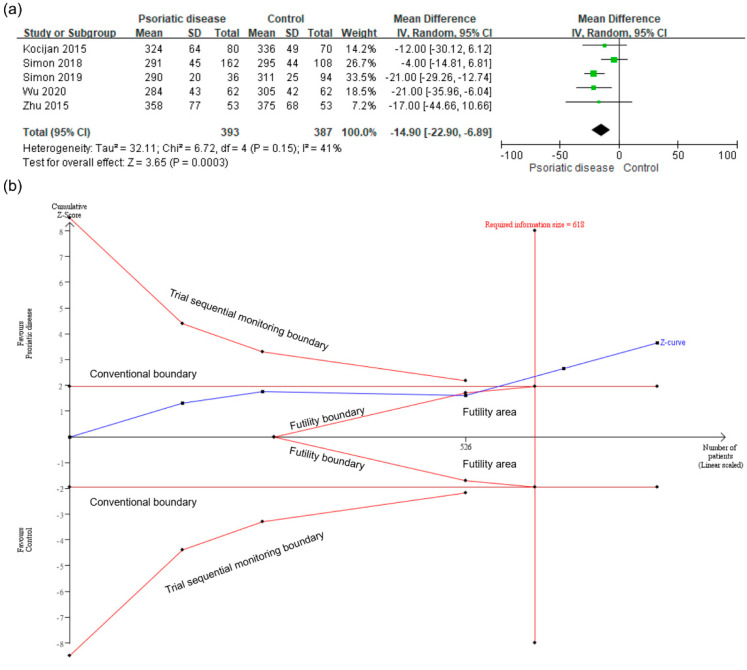
(**a**) meta-analysis of the mean difference in the average volumetric bone mineral density (vBMD) in psoriatic patients and controls. (**b**) trial sequential analysis of the average vBMD. CI, confidence interval; IV, inverse variance; SD, standard deviation.

The meta-analysis of five studies demonstrated that patients with psoriatic disease showed a lower average vBMD than controls (MD −14.90; 95% CI −22.90 to −6.89; TSA-adjusted CI −23.77 to −6.03; I^2^ = 41%; Figure 2a). An IS was calculated based on α = 5% (two-sided) and β = 10%. The cumulative Z-curve crossed the monitoring boundary of TSA and reached the required IS of 618, making the results conclusive (Figure 2b).

**Figure 3:**
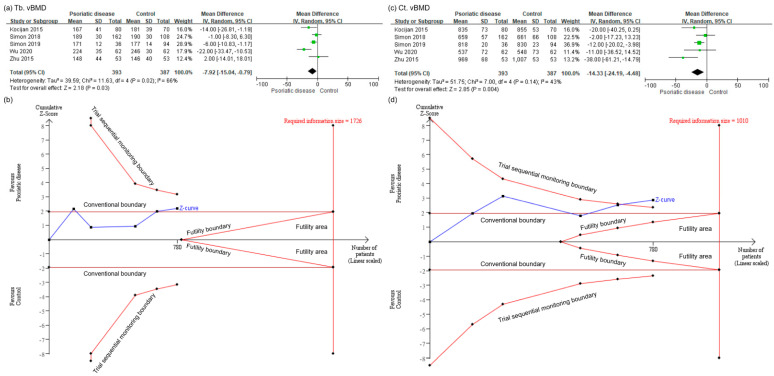
(**a**) meta-analysis of the mean difference in trabecular volumetric bone mineral density (vBMD) in psoriatic patients and controls. (**b**) trial sequential analysis of trabecular vBMD. (**c**) meta-analysis of the mean difference in cortical vBMD in psoriatic patients and controls. (**d**) trial sequential analysis of cortical vBMD. CI, confidence interval; Ct, cortical; IV, inverse variance; SD, standard deviation; Tb, trabecular.

Results pertaining to secondary outcomes also indicated that patients with psoriatic disease showed lower trabecular vBMD (MD −7.92; 95% CI −15.04 to −0.79; I^2^ = 66%; Figure 3a) and cortical vBMD (MD −14.33; 95% CI −24.19 to −4.48; I^2^ = 43%; Figure 3c) than controls. However, after TSA adjustment, the cumulative Z-curve of trabecular vBMD neither crossed the monitoring boundary of TSA nor reached the required IS of 1726 (Figure 3b). The TSA-adjusted CI of MD in trabecular vBMD was −19.43 to 3.59, refuting our previous analysis. Thus, we were unable to make conclusions regarding trabecular vBMD in psoriatic patients. Moreover, the cumulative Z-curve of cortical vBMD crossed the monitoring boundary of TSA but did not reach the required IS of 1010 (Figure 3d). Since the sample size was small, a conclusion could also not be achieved regarding cortical vBMD in patients with psoriatic disease.

### 3.5. Subgroup Meta-Analysis

As shown in Table 2, psoriatic patients recruited in Asia and those whose vBMDs were measured at the distal radius exhibited a lower average vBMD than controls. Thus, geographic locations and measuring sites might be study-level factors that could influence the pooled results.

## 4. Discussion

To the best of our knowledge, the present systematic review and meta-analysis is the first to investigate the association of psoriatic disease with vBMD measured by HR-pQCT. Our findings demonstrated that patients with psoriatic disease had a lower average vBMD than controls. The effect is significant in studies conducted in Asia and in those whose vBMD was measured at the distal radius. While psoriatic patients showed lower trabecular vBMD and cortical vBMD than controls, the results of TSA indicated that the sample sizes were insufficient to draw conclusions.

A previous analysis concluded that psoriatic patients have a higher fracture risk than non-psoriatic controls [11]. Furthermore, lower vBMD in psoriatic patients may be related to lower bone quality and contributes to the higher risk of fracture [35]. However, the authors were unsure about whether the vBMD was higher or lower in psoriatic patients. Thus, the present study was carried out to address this knowledge gap, and the results were consistent with their hypothesis.

The pathogenesis of imbalanced bone remodeling in psoriasis has not been well understood [1]. In recent studies, the interleukin (IL)-23/IL-17 pathway is suggested to have important role in psoriatic bone remodeling [36,37]. Overexpressed pro-inflammatory cytokines in which multiple signaling pathways are involved may promote pathologic bone resorption [38]. Moreover, genetic predisposition and environmental factors may induce immune dysregulation, causing alterations in osteocyte activity [39,40].

In the subgroup analysis, geographic locations and anatomical sites of measurement were identified as potential effect modifiers of vBMD in psoriatic patients. A lower vBMD was observed in studies that were conducted in Asia than in those conducted in Europe [30,31]. Previous inter-ethnic comparisons have noted that Asian patients have a lower aBMD and vBMD than Caucasians [41,42]. After adjustment for bone size, weight, and height, a lower vBMD was only seen in postmenopausal women and men aged less than 50 years [41]. Nevertheless, the mechanism behind this phenomenon was unclear [42].

As seen in Table 2, vBMD measured at the distal radius was lower in psoriatic patients than in controls, which indicated another potential effect modifier. This result supports a previous finding that vBMD at peripheral sites is a robust independent predictor of osteoporotic fracture [43]. Bone erosions starting at the capsular insertion in the pathogenesis of psoriatic disease may explain our observation [10,44].

A study suggested that declines in aBMD may only explain 70% of bone strength impairment [45], and some fragility fractures occur in patients with normal aBMD or osteopenia. To date, the application of HR-pQCT has provided insights into the bone microstructure of several rheumatic diseases other than psoriasis. Lower vBMD has been shown in diseases such as rheumatoid arthritis [44], ankylosing spondylitis [46], and systemic lupus erythematosus [47].

A major strength of this study is that the authors compiled up-to-date evidence and have provided study direction for future studies. The heterogeneity across the enrolled studies was not substantial. However, the results need to be interpreted in light of their limitations. First, the sample size of the enrolled studies was still small. Despite the application of TSA to avoid type I error, the authors could only conclude on the relationship between average vBMD and psoriatic disease. Second, the studies were conducted in only three countries. Larger studies with more diverse populations may be beneficial to enhance the external validity of the conclusion. Third, the conclusions were based on clinical data from observational studies. The results could only explain the association of psoriatic disease with lower vBMD. Future studies appear necessary to elucidate the molecular and immunological pathogeneses of these conditions.

## 5. Conclusions

In the present study, given the limited evidence, patients with psoriatic disease had a lower average vBMD than controls. The differences in geographic location and anatomical site of measurement may play important roles in the vBMD of psoriatic patients. Further research is warranted to clarify the underlying pathophysiology.

## Figures and Tables

**Table 1:** Characteristics of included studies.

First Author, Year	Study Design	Country	No. of Participants		Age (Years) Mean ± SD	Sex (Female %)	BMI (kg/m^2^) Mean ± SD	Disease Duration (Years) Mean ± SD	Drug * Use	vBMD Measurement	
										Device ^#^	Site
Kocijan, 2015	Cross-sectional	Germany	Psoriatic disease Control	80 70	50.9 ± 12.8 51.5 ± 13.5	52.0 50.0	27.9 ± 5.1 26.2 ± 4.8	8.0 ± 7.3 NA	Yes	HR-pQCT	DR
Simon, 2018	Cross-sectional	Germany	Psoriatic disease Control	162 108	50.6 ± 12. 349.8 ± 16.6	46.6 60.2	29.3 ± 6.0 24.5 ± 3.7	18.4 ± 13.7 NA	Yes	HR-pQCT	MCH
Simon, 2019	Case-control	Austria, Germany	Psoriatic disease Control	36 94	46.7 56.1	36.1 28.7	28.0 27.8	NA	No	HR-pQCT	DR
Wu, 2020	Cross-sectional	China	Psoriatic disease Control	62 62	52.94 ± 11.5 50.8 ± 9.3	45.2 50.0	25.2 ± 4.1 24.0 ± 3.0	13.1 ± 7.2	Yes	HR-pQCT	MCH
Zhu, 2015	Cross-sectional	China	Psoriatic disease Control	53 53	53.1 ± 8.9 52.9 ± 9.4	54.7 54.7	25.2 ± 3.7 24.2 ± 3.0	14.0 ± 7.2	Yes	HR-pQCT	DR

* We demonstrated potential osteoporotic/anti-osteoporotic drug usage, such as systemic corticosteroid, methotrexate, or anti-TNF alpha biologic agents. ^#^ HR-pQCT manufacturer was listed if available. BMI, body mass index; DR, distal radius; HR-pQCT, high-resolution peripheral quantitative computed tomography; MCH, metacarpal head; NA, not applicable; No., number; PsA, psoriatic arthritis; SD, standard deviation; vBMD, volumetric bone mineral density.

**Table 2:** Subgroup analysis.

Subgroups	No. of Studies	MD in Average vBMD	95% CI	TSA Adjusted 95% CI	I^2^ (%)
Overall	5	−14.90	−22.90 to −6.89 *	−23.77 to −6.03 *	41
Geographic location					
Europe	3	−12.82	−24.64 to −1.00 *	−29.97 to 4.33	67
Asia	2	−20.09	−33.25 to −6.93 *	−34.65 to −5.53 *	0
Anatomical site					
Distal radius	3	−19.28	−26.54 to −12.03 *	−26.69 to −11.88 *	0
Metacarpal head	2	−11.68	−28.26 to 4.90	−79.36 to 56.00	69

* *p* < 0.05, indicated statistically significant. CI, confidence interval; MD, mean difference; No., number; TSA, trial sequential analysis; vBMD, volumetric bone mineral density.

## Data Availability

Data were accessed via the Electronic Resources Federated Search System of Buddhist Tzu Chi Medical Foundation Library. Review Manager 5.4.1 (Cochrane Collaboration, Oxford, England) was used to conduct the present meta-analysis.

## Supplementary Materials

The following are available online at https://www.mdpi.com/article/10.3390/healthcare9081056/s1, Table S1. Detailed search strategy modified to accommodate different databases. Figure S1. Summary of risk of bias assessment.

## References

[1] Armstrong A.W., Read C. (2020). Pathophysiology, clinical presentation, and treatment of psoriasis: A review. JAMA.

[2] Parisi R., Iskandar I.Y., Kontopantelis E., Augustin MGriffiths C.E., Ashcroft D.M. (2020). National, regional, and worldwide epidemiology of psoriasis: Systematic analysis and modelling study. BMJ.

[3] Ritchlin C.T., Colbert R.A., Gladman D.D. (2017). Psoriatic arthritis. N. Engl. J. Med..

[4] Bilal J., Malik S.U., Riaz I.B., Kurtzman D.J.B. (2018). Psoriasis and psoriatic spectrum disease: A primer for the primary care physician. Am. J. Med..

[5] Stuart P.E., Nair R.P., Tsoi L.C., Tejasvi T., Das S., Kang H.M., Ellinghaus E., Chandran V., Callis-Duffin K., Ike R. (2015). Genome-wide association analysis of psoriatic arthritis and cutaneous psoriasis reveals differences in their genetic architecture. Am. J. Hum. Genet..

[6] Sakkas L.I., Bogdanos D.P. (2017). Are psoriasis and psoriatic arthritis the same disease? The IL-23/IL-17 axis data. Autoimmun. Rev..

[7] Okhovat J.P., Ogdie A., Reddy S.M., Rosen C.F., Scher J.U., Merola J.F. (2017). Psoriasis and Psoriatic Arthritis Clinics Multicenter Advancement Network Consortium (PPACMAN) survey: Benefits and challenges of combined rheumatology-dermatology clinics. J. Rheumatol..

[8] Savage L., Tinazzi I., Zabotti A., Laws P.M., Wittmann M., McGonagle D. (2020). Defining pre-clinical psoriatic arthritis in an integrated dermato-rheumatology environment. J. Clin. Med..

[9] Sirufo M.M., De Pietro F., Bassino E.M., Ginaldi L., De Martinis M. (2020). Osteoporosis in skin diseases. Int. J. Mol. Sci..

[10] Paine A., Ritchlin C. (2018). Altered bone remodeling in psoriatic disease: New insights and future directions. Calcif. Tissue Int..

[11] Chen T.L., Lu J.W., Huang Y.W., Wang J.H., Su K.Y. (2020). Bone mineral density, osteoporosis, and fracture risk in adult patients with psoriasis or psoriatic arthritis: A systematic review and meta-analysis of observational studies. J. Clin. Med..

[12] Ott S.M. (2016). Bone strength: More than just bone density. Kidney Int..

[13] Torres-del-Pliego E., Vilaplana L., Güerri-Fernández R., Diez-Pérez A. (2013). Measuring bone quality. Curr. Rheumatol. Rep..

[14] Geusens P., Chapurlat R., Schett G., Ghasem-Zadeh A., Seeman E., De Jong J., Bergh J.V.D. (2014). High-resolution in vivo imaging of bone and joints: A window to microarchitecture. Nat. Rev. Rheumatol..

[15] Lespessailles E., Ibrahim-Nasser N., Toumi H., Chapurlat R. (2018). Contribution of high resolution peripheral quantitative CT to the management of bone and joint diseases. Joint Bone Spine.

[16] Nishiyama K.K., Macdonald H.M., Buie H.R., Hanley D.A., Boyd S.K. (2010). Postmenopausal women with osteopenia have higher cortical porosity and thinner cortices at the distal radius and tibia than women with normal aBMD: An in vivo HR-pQCT study. J. Bone Miner. Res..

[17] Nishiyama K.K., Macdonald H.M., Hanley D.A., Boyd S.K. (2013). Women with previous fragility fractures can be classified based on bone microarchitecture and finite element analysis measured with HR-pQCT. Osteoporos Int..

[18] Whittier D., Boyd S., Burghardt A., Paccou J., Ghasem-Zadeh A., Chapurlat R., Engelke K., Bouxsein M. (2020). Guidelines for the assessment of bone density and microarchitecture in vivo using high-resolution peripheral quantitative computed tomography. Osteoporos Int..

[19] Page M.J., McKenzie J.E., Bossuyt P.M., Boutron I., Hoffmann T.C., Mulrow C.D., Shamseer L., Tetzlaff J.M., Akl E.A., Brennan S.E. (2021). The PRISMA 2020 statement: An updated guideline for reporting systematic reviews. BMJ.

[20] Stroup D.F., Berlin J.A., Morton S.C., Olkin I., Williamson G.D., Rennie D., Moher D., Becker B.J., Sipe T.A., Thacker S.B. (2000). Meta-analysis of observational studies in epidemiology: A proposal for reporting. Meta-analysis Of Observational Studies in Epidemiology (MOOSE) group. JAMA.

[21] Seo H.J., Kim S.Y., Lee Y.J., Jang B.H., Park J.E., Sheen S.S., Hahn S.K. (2016). A newly developed tool for classifying study designs in systematic reviews of interventions and exposures showed substantial reliability and validity. J. Clin. Epidemiol..

[22] Wells G.A., Shea B.J., O’Connell D., Peterson J., Welch V., Losos M., Tugwell P. The Newcastle-Ottawa Scale (NOS) for Assessing the Quality of Non-Randomised Studies in Meta-Analyses. http://www.ohri.ca/programs/clinical_epidemiology/oxford.htm.

[23] Higgins J.P.T., Thomas J., Chandler J., Cumpston M., Li T., Page M.J., Welch V.A. Cochrane Handbook for Systematic Reviews of Interventions Version 6.2 (updated February 2021). www.training.cochrane.org/handbook.

[24] Higgins J.P., Thompson S.G., Deeks J.J., Altman D.G. (2003). Measuring inconsistency in meta-analyses. BMJ.

[25] Brok J., Thorlund K., Gluud C., Wetterslev J. (2008). Trial sequential analysis reveals insufficient information size and potentially false positive results in many meta-analyses. J. Clin. Epidemiol..

[26] Shah A., Smith A.F. (2020). Trial sequential analysis: Adding a new dimension to meta–analysis. Anaesthesia.

[27] Kocijan R., Englbrecht M., Haschka J., Simon D., Kleyer A., Finzel S., Kraus S., Resch H., Muschitz C., Engelke K. (2015). Quantitative and qualitative changes of bone in psoriasis and psoriatic arthritis patients. J. Bone Miner. Res..

[28] Simon D., Kleyer A., Englbrecht M., Stemmler F., Simon C., Berlin A., Kocijan R., Haschka J., Hirschmann S., Atreya R. (2018). A comparative analysis of articular bone in large cohort of patients with chronic inflammatory diseases of the joints, the gut and the skin. Bone.

[29] Simon D., Haschka J., Muschitz C., Kocijan A., Baierl A., Kleyer A., Schett G., Kapiotis S., Resch H., Sticherling M. (2020). Bone microstructure and volumetric bone mineral density in patients with hyperuricemia with and without psoriasis. Osteoporos Int..

[30] Wu D., Griffith J.F., Lam S.H.M., Wong P., Yue J., Shi L., Li E.K., Cheng I.T., Li T.K., Hung V.W. (2020). Comparison of bone structure and microstructure in the metacarpal heads between patients with psoriatic arthritis and healthy controls: An HR-pQCT study. Osteoporos Int..

[31] Zhu T.Y., Griffith J.F., Qin L., Hung V.W.Y., Fong T.-N., Au S.-K., Kwok A.W., Leung P.-C., Li E.K., Tam L.-S. (2014). Density, structure, and strength of the distal radius in patients with psoriatic arthritis: The role of inflammation and cardiovascular risk factors. Osteoporos Int..

[32] Buckley L., Humphrey M.B. (2018). Glucocorticoid-induced osteoporosis. N. Engl. J. Med..

[33] Orsolini G., Fassio A., Rossini M., Adami G., Giollo A., Caimmi C., Idolazzi L., Viapiana O., Gatti D. (2019). Effects of biological and targeted synthetic DMARDs on bone loss in rheumatoid arthritis. Pharmacol. Res..

[34] Kawai V.K., Stein C.M., Perrien D.S., Griffin M.R. (2012). Effects of anti-tumor necrosis factor α agents on bone. Curr. Opin. Rheumatol..

[35] Darelid A., Ohlsson C., Rudäng R., Kindblom J.M., Mellström D., Lorentzon M. (2010). Trabecular volumetric bone mineral density is associated with previous fracture during childhood and adolescence in males: The GOOD study. J. Bone Miner. Res..

[36] Ebihara S., Date F., Dong Y., Ono M. (2015). Interleukin-17 is a critical target for the treatment of ankylosing enthesitis and psoriasis-like dermatitis in mice. Autoimmunity.

[37] Adamopoulos I.E., Suzuki E., Chao C.-C., Gorman D., Adda S., Maverakis E., Zarbalis K., Geissler R., Asio A., Blumenschein W.M. (2015). IL-17A gene transfer induces bone loss and epidermal hyperplasia associated with psoriatic arthritis. Ann. Rheum Dis.

[38] Dalbeth N., Pool B., Smith T., Callon K.E., Lobo M., Taylor W.J., Jones P.B., Cornish J., McQueen F.M. (2010). Circulating mediators of bone remodeling in psoriatic arthritis: Implications for disordered osteoclastogenesis and bone erosion. Arthritis Res. Therapy..

[39] Bowes J., Ashcroft J., Dand N., Jalali-Najafabadi F., Bellou E., Ho P., Marzo-Ortega H., Helliwell P.S., Feletar M., Ryan A. (2017). Cross-phenotype association mapping of the MHC identifies genetic variants that differentiate psoriatic arthritis from psoriasis. Ann. Rheum. Dis..

[40] Raimondo A., Lembo S., Di Caprio R., Donnarumma G., Monfrecola G., Balato N., Ayala F., Balato A. (2017). Psoriatic cutaneous inflammation promotes human monocyte differentiation into active osteoclasts, facilitating bone damage. Eur. J. Immunol..

[41] Lau E.M., Lynn H., Woo J., Melton L.J. (2003). Areal and volumetric bone density in Hong Kong Chinese: A comparison with Caucasians living in the United States. Osteoporos Int..

[42] Durdin R., Parsons C.M., Dennison E., Harvey N.C., Cooper C., Ward K. (2020). Ethnic differences in bone microarchitecture. Curr. Osteoporos Rep..

[43] Langsetmo L., Peters K.W., Burghardt A.J., Ensrud K., Fink H.A., Cawthon P.M., Cauley J.A., Schousboe J.T., Barrett-Connor E., Orwoll E.S. (2018). Volumetric bone mineral density and failure load of distal limbs predict incident clinical fracture independent of FRAX and clinical risk factors among older men. J. Bone Miner. Res..

[44] Villani A.P., Boutroy S., Coutisson C., Carlier M.C., Barets L., Marotte H., Richert B., Chapurlat R.D., Jullien D., Confavreux C.B. (2020). Distal phalangeal bone erosions observed by HR-pQCT in patients with psoriatic onycholysis. Rheumatology.

[45] Jin S., Li M., Wang Q., Zeng X., Xia W., Yu W., Guan W., Hsieh E. (2021). Bone mineral density and microarchitecture among Chinese patients with rheumatoid arthritis: A cross-sectional study with HRpQCT. Arthritis Res. Ther.

[46] Klingberg E., Lorentzon M., Göthlin J., Mellström D., Geijer M., Ohlsson C., Atkinson E.J., Khosla S., Carlsten H., Forsblad-d’Elia H. (2013). Bone microarchitecture in ankylosing spondylitis and the association with bone mineral density, fractures, and syndesmophytes. Arthritis Res. Ther..

[47] Tang X.L., Qin L., Kwok A.W., Zhu T.Y., Kun E.W., Hung V.W., Griffith J.F., Leung P.C., Li E.K., Tam L.S. (2013). Alterations of bone geometry, density, microarchitecture, and biomechanical properties in systemic lupus erythematosus on long-term glucocorticoid: A case-control study using HR-pQCT. Osteoporos Int..

