# Supplementary material for: Volumetric Bone Mineral Density Measured by HR-pQCT in Patients with Psoriasis or Psoriatic Arthritis: A Systematic Review and Meta-Analysis with Trial Sequential Analysis
Source: Healthcare (Basel). 2021 Aug 17;9(8):1056. doi: 10.3390/healthcare9081056 (PMC8393585; doi:10.3390/healthcare9081056)
Supplement: Supplementary file 1 [file healthcare-09-01056-s001.zip › healthcare-1293687-supplementary.pdf]

Supplementary Materials

Table S1. Detailed search strategy modified to accommodate different databases.

Database	Search date	Search term		Results
PubMed	2021/03/02	Strategy	#1 AND #2	425
		#1	((Psoriasis [mesh] OR (Psorias*) OR (Pustulosis of Palms and Soles) OR (Pustulosis Palmaris et Plantaris) OR (Palmoplantaris Pustulosis) OR (Pustular Psoriasis of Palms and Soles)) OR (Arthritis, Psoriatic [mesh] OR (Psoriasis, Arthritic) OR (Psoriatic Arthriti*) OR (Psoriasis Arthropathica) OR (Psoriatic Arthropath*))	
		#2	(Bone Density [mesh] OR (Bone Densit*) OR (Bone Mineral Densit*) OR (Bone Mineral Content*) OR (BMD) OR (Bone Mass Density) OR (microstructure) OR (Bone microstructure) OR (Osteoporosis)	
Ovid Embase	2021/03/03	Strategy	(#1 or #2) and (#3 or #4 or #5 or #6)	1851
		#1	exp Psoriasis/ or Psoriasis.mp. [mp=title, abstract, original title, name of substance word, subject heading word, floating sub-heading word, keyword heading word, organism supplementary concept word, protocol supplementary concept word, rare disease supplementary concept word, unique identifier, synonyms]	
		#2	((((palmoplantaris pustulosis or psoriases or psoriasis or pustular psoriasis of palms) and soles) or pustulosis palmaris et plantaris or pustulosis of palms) and soles).mp.	
		#3	(bone density or BMD).ti,ab. or Bone Density/	
		#4	bone mineral density.ti,ab.	
		#5	(bone mineral content or BMC).ti,ab.	
		#6	(bone adj (mass or strength or loss or accret$ or remodel$ or resorp$)).ti,ab.	
Web of Science	2021/03/05	Strategy	#1 AND #2	254
		#1	TS=((Psoriasis)) OR ALL=(((Psorias*) OR (Pustulosis of Palms and Soles) OR (Pustulosis Palmaris et Plantaris) OR (Palmoplantaris	

			Pustulosis) OR (Pustular Psoriasis of Palms and Soles))) OR TS=((Arthritis, Psoriatic)) OR ALL=(((Psoriasis, Arthritic) OR (Psoriatic Arthriti*) OR (Psoriasis Arthropathica) OR (Psoriatic Arthropath*))) OR TS=(((psoria* arthrit*) OR (psoria* arthropath*)))	
		#2	TS=((Bone Density)) OR ALL=(((bone densit*) OR (bone mineral content*) OR (bone mineral densit*) OR (Bone Mass Density) OR (BMD))) OR TS=((Osteoporosis)) OR ALL=(((age related osteoporos*) OR (bone loss* age related) OR (osteoporos*) OR (osteoporosis, involutional) OR (post-traumatic osteoporos*) OR (senile osteoporos*))) OR ALL=(((microstructure) OR (Bone microstructure)))	
Cochrane Library	2021/03/05	Strategy	#1 AND #2	83
		#1	(Psoriasis OR (Psorias*) OR (Pustulosis of Palms and Soles) OR (Pustulosis Palmaris et Plantaris) OR (Palmoplantaris Pustulosis) OR (Pustular Psoriasis of Palms and Soles)) OR (Arthritis, Psoriatic) OR (Psoriasis, Arthritic) OR (Psoriatic Arthriti*) OR (Psoriasis Arthropathica) OR (Psoriatic Arthropath*)	
		#2	(Bone Density) OR (Bone Densit*) OR (Bone Mineral Content*) OR (BMD) OR (Bone Mass Densit*) OR (microstructure) OR (Bone microstructure) OR (Osteoporosis)	

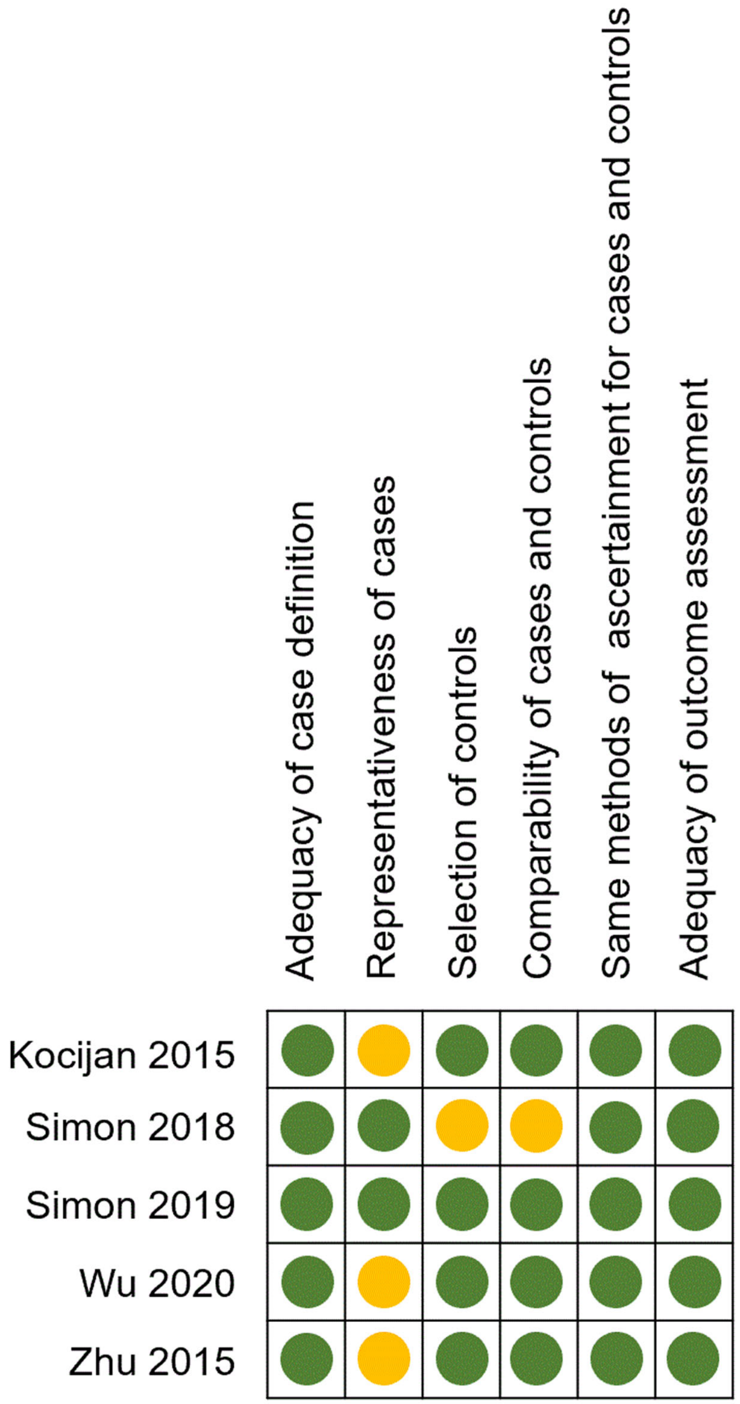

Figure S1. Summary of risk of bias assessment.
